# Sunset Yellow Confined in Curved Geometry: A Microfluidic Approach

**DOI:** 10.1021/acs.langmuir.3c00275

**Published:** 2023-04-19

**Authors:** Caterina Maria Tone, Alessandra Zizzari, Lorenza Spina, Monica Bianco, Maria Penelope De Santo, Valentina Arima, Riccardo Cristoforo Barberi, Federica Ciuchi

**Affiliations:** † Physics Department, University of Calabria, Ponte Bucci, cubo 31C, 87036 Arcavacata di Rende, CS, Italy; ‡ CNR-Nanotec, c/o Physics Department, University of Calabria, Ponte Bucci, cubo 31C, 87036 Arcavacata di Rende, CS, Italy; § CNR NANOTEC − Institute of Nanotechnology, c/o Campus Ecotekne, University of Salento, via Monteroni, 73100 Lecce, Italy

## Abstract

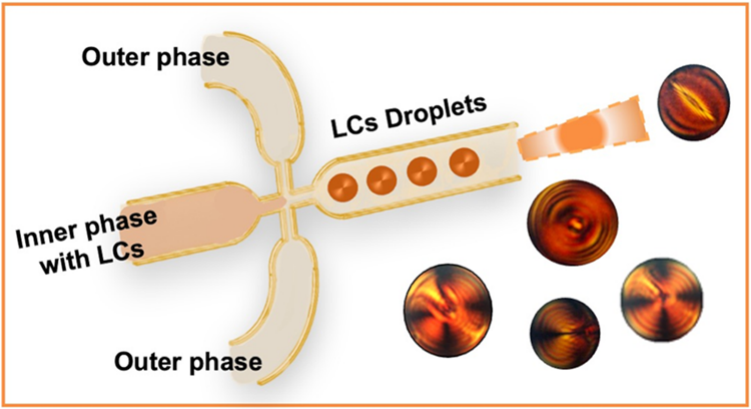

The behavior of lyotropic chromonic liquid crystals (LCLCs) in confined environments is an interesting research field that still awaits exploration, with multiple key variables to be uncovered and understood. Microfluidics is a highly versatile technique that allows us to confine LCLCs in micrometric spheres. As microscale networks offer distinct interplays between the surface effects, geometric confinement, and viscosity parameters, rich and unique interactions emerging at the LCLC–microfluidic channel interfaces are expected. Here, we report on the behavior of pure and chiral doped nematic Sunset Yellow (SSY) chromonic microdroplets produced through a microfluidic flow-focusing device. The continuous production of SSY microdroplets with controllable size gives the possibility to systematically study their topological textures as the function of their diameters. Indeed, doped SSY microdroplets produced via microfluidics, show topologies that are typical of common chiral thermotropic liquid crystals. Furthermore, few droplets exhibit a peculiar texture never observed for chiral chromonic liquid crystals. Finally, the achieved precise control of the produced LCLC microdroplets is a crucial step for technological applications in biosensing and anticounterfeiting.

## Introduction

In recent years, thermotropic liquid crystals (LCs) have been confined and studied in droplets and shells showing complex topological defects strongly related to their intrinsic anisotropy.^1−5^

Lyotropic chromonic liquid crystals are a special class of lyotropic LCs that are formed by anisotropic assemblies of water-soluble disk-shaped molecules that have an aromatic core surrounded by ionic groups. Among LCLCs, there are DNA and its bases, disodium cromoglycate (DSCG), a commonly used drug, and sunset yellow (SSY), a dye used in the food industry.^6,7^ Unlike lyotropic LCs, LCLCs do not form micelles; rather, they stack up as linear aggregates, held together by noncovalent interactions, which lead to a self-assembled nematic phase or a columnar phase with a hexagonal arrangement possessing unique optical properties.^8−11^ The weak interaction forces driving the self-assembled phase formation make LCLCs highly responsive to external stimuli (temperature, concentration, pH, ionic content, etc.) and geometric constraints, thus conferring them distinctive properties such as negative birefringence and a large anisotropy in the elastic constants. Moreover, owing to biocompatibility and anisotropic properties, LCLCs have been explored in biological applications such as drug delivery^12^ and optical biosensing^13^ and in technological applications.^14^ Curved confinement can trigger unconventional effects on LCLCs because their alignment direction sufficiently anchored at the boundary will be transmitted into the bulk in the form of an orientation field deformation. The latter leads to the generation of new structures with controllable topological defects or dislocations. Recently, few research groups have studied the confinement-induced reflection symmetry breaking of nematic LCLCs in curved geometries like tactoids, microspheres, or cylinders.^15−19^ Cholesteric LCLCs confined in microspheres have been studied as well, the chirality in some cases is native,^20^ while in others, it can be induced by doping the nematic phase with suitable molecules, for example, amino acids.^21,22^ Microspheres are obtained by emulsification, i.e., through the mechanical agitation of a mixture based on an immiscible matrix and the LCLC phase.^23,24^

Despite the large number of microspheres produced by this method, it is not possible to achieve fine control of their size, which plays a fundamental role in their final optical texture. This is important for applications (i.e., anticounterfeiting or biosensing) in which the production of precise size distributions of microstructures with particular optical features is the prerequisite for the correct functionality of the device.^25,26^ The size control can be achieved using a microfluidic approach by opportunely tuning the flow conditions and surfactant concentration.

Nowadays, advancement in microfabrication techniques of various materials has enabled the precise design and modulation of structural features, pressure, and boundary conditions, with the consequent conspicuous development of microfluidic devices applied to biological and materials technology.^27−31^ Microfluidics has the unique advantages of the continuous flow production of multifunctional materials with accurate control of structural properties^32−37^ and allows distinct interplays between the surface effects, geometric confinement, viscosity parameters, and interfacial phenomena^38^ with promising applications in the LCLCs field. Recent progress in liquid crystal microfluidics has demonstrated how hydrodynamics, in combination with surface interactions, geometric confinement, and flow modulation can be harnessed to generate topological structures with potential for novel applications.^39,40^ Previous works have shown that interesting phenomena are observed by precisely tuning the flow of nematic LCs, the confinement conditions in microchannels, and the wettability of channel walls.^41−44^ On the other hand, studies on the microfluidic generation of droplets embodying a chromonic liquid crystal and an isotropic component are still rare. Only a few works report on the production through microfluidics of chromonic cholesteric droplets of cellulose nanocrystals (CNCs)^45,46^ and pure SSY.^47^ In the last case, the authors employed a microfluidic system that did not make use of electromechanical injection, a crucial point for systems in which viscosity may increase, as, for example, in the case of chromonic materials doped with chiral moieties. To the best of our knowledge, the control of microdroplets’ chromonic diameter in microfluidic devices has never been reported.

In the present work, the microfluidic generation chromonic SSY microdroplets with precise size and optical properties obtained by tuning the flow conditions is described. We were able to produce via microfluidics, microdroplets with rich and unique topologies, such as those observed for the classic nematic and cholesteric thermotropic LCs, as well as microdroplets with topologies never observed before in chiral chromonic droplets. Initially, a description of the strategies used to obtain stable droplets with defined optical textures will be provided. Then, a polarized light optical microscopy study will be presented to gain information on the director field configuration inside microspheres.

The production of well-defined size-dependent optical textures is the first crucial step toward the use of chromonic microspheres for practical applications. In particular, it has been recently demonstrated that the optical topology of microspheres degrades irreversibly over time due to temperature.^48^ Degradation can be slowed down by keeping the samples at 5 °C and can be avoided if samples are stored at −18 °C. This paves the way for the fabrication of new biocompatible time–temperature indicators that can be used to monitor the cold food chain.

## Results and Discussion

**Figure 1:**
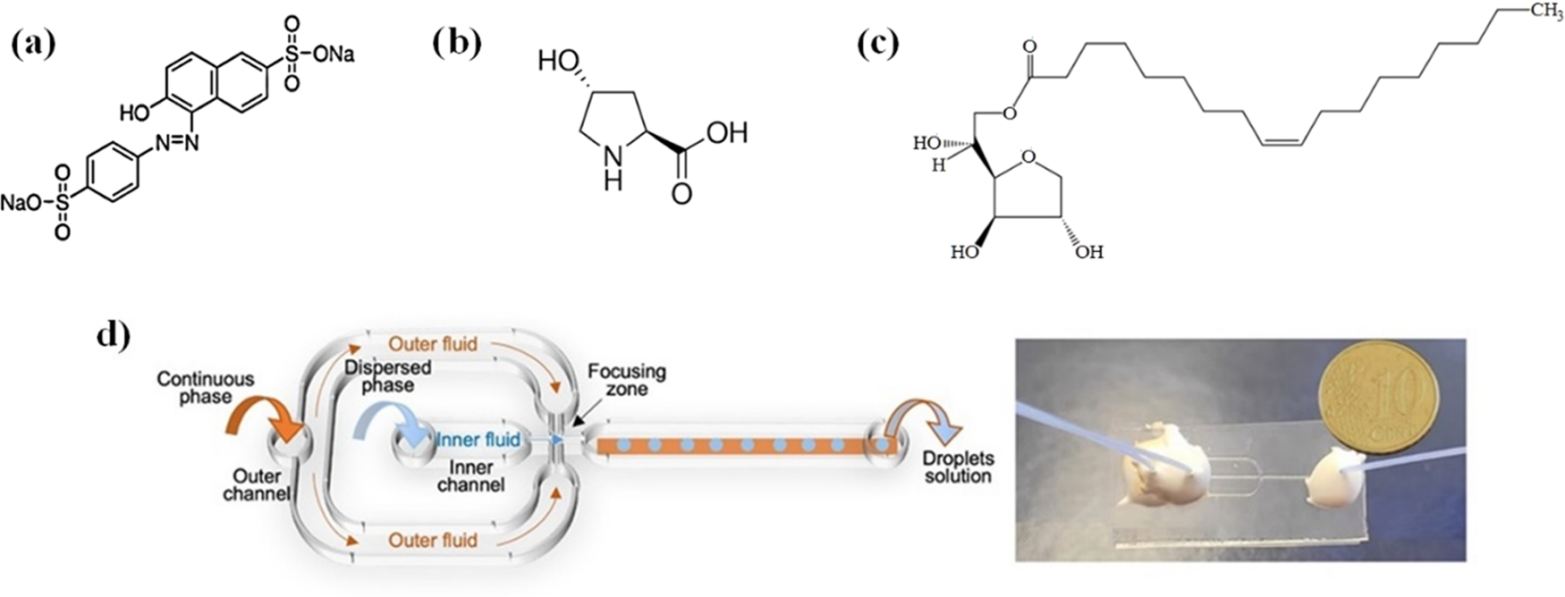
Molecular structures of (a) SSY, (b) *trans*-4-hydroxy-l-proline, and (c) Span80. In (d) on the left, flow-focusing device sketch: the continuous phase (paraffin oil) and the dispersed phase (SSY aqueous solution) were injected with programmable syringe pumps in the outer and inner channels, respectively. The droplets were generated at the focusing zone and then collected for the SSY microspheres analysis. A picture of the FF device used for the experiments is shown on the right.

The molecular formula of SSY is shown in Figure 1a. In this work, both pure SSY solution, in the nematic and isotropic phase, and SSY mixtures doped with *trans*-4-hydroxy-l-prolyne (Trans-Hyp, see Figure 1b), which acts as a chiral agent, were analyzed. Microdroplets of all of the solutions have been produced by a microfluidic flow-focusing device (Figure 1d) using a nonionic surfactant (Span80, see Figure 1c) dissolved in the paraffin oil phase. The glass walls of the device are covered by a silane layer that enhances the surface hydrophobicity, increasing the affinity of the oil phase for the walls and decreasing that of the aqueous phase, as shown by the water contact angle measurement of the clean and functionalized glass substrate, reported in Table 2.

### Microdroplet Production

The dispersed (aqueous) phase was injected in the inner microchannel and then squeezed by continuous (oil) phase flow injected by the outer microchannel. In this geometry, the symmetric shear generated by the continuous phase on the dispersed phase allows a highly controlled and stable generation of droplets.^36,49,50^ Indeed, the central stream of the dispersed phase becomes so narrow to break into droplets,^36,51^ at different possible flow regimes (mainly squeezing, dripping, jetting, and threading regime),^52^ depending on the delicate balance between the capillary number Ca (defined as the ratio of viscous force to interfacial tension) and the volumetric flow rate ratio (FRR) between the outer and inner fluids.

First, we identified the flow conditions and the surfactant amount necessary to induce the inner fluid to break into stable droplets for a 7% wt SSY concentration, which corresponds to the isotropic phase of the SSY liquid crystal solution. The surfactants are a facet of the microfluidic technique and are not necessary to produce LCLC droplets by mechanical shaking.^24^ Indeed, in the microfluidic environments, the surfactant plays a fundamental role^49^ in inducing the formation of the droplets as the surfactant molecules tend to assemble at the droplet interface, exposing their polar heads toward the water phase and their nonpolar tails toward the oil phase. In our setup, the breaking of the stream into droplets for a 4% wt Span80 concentration was observed.

**Figure 2:**
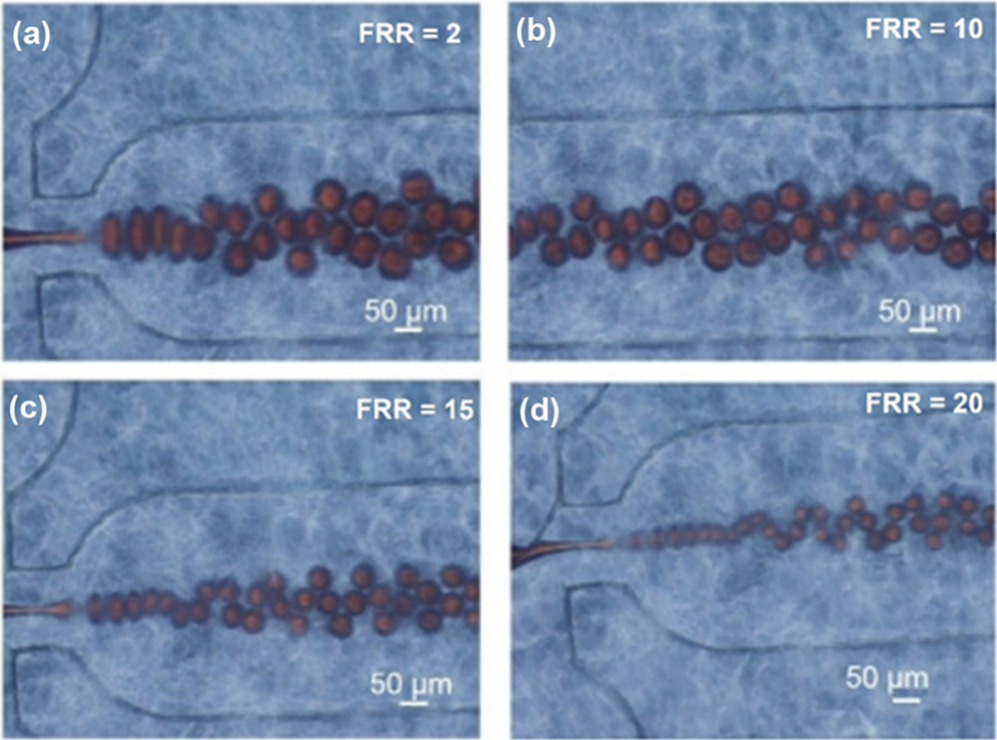
Images of the FF device generating droplets of SSY 7% wt in water (red solution injected from the inner channel of Figure 1d) dispersed into the paraffin oil in the presence of Span80 4% wt (flowing from the outer channel of Figure 1d). Droplets of different sizes are produced at (a) FRR = 2, (b) FRR = 10, (c) FRR = 15, and (d) FRR = 20.

At this concentration, the diameter of the droplets can be varied by tuning the flow ratios of the two fluids, in the order of tens of μL/min for both fluids, obtaining a controlled diameter range of tens of microns (see Figure 2). In Figure S1, an example of the diameter distribution of isotropic droplets is shown.

For an FRR of 2, SSY droplets presented a diameter of 50 μm with a tendency to coalesce inside the channel. When the flow ratios increased up to 10, droplet diameters scaled around 30 μm, while for an FRR of 15, the shear forces generated at the focusing zone allowed the formation of a narrow thread of the inner fluid and its breakage into monodisperse droplets with diameters much smaller than the orifice width (around 25 μm). The decrease in the droplet size with the increase in FRR is typical of systems with low viscosity contrast of fluids.^53^ Furthermore, we occasionally observed a jet of small monodisperse droplets (Figure 2c,d), already seen by Anna et al.^54^ for a different liquid system. This behavior is due to the Rayleigh plateau instability occurring when a fluid is forced to travel rapidly through a microchannel.^52,55,56^ Interestingly, in this case, the droplet downstream arrangement assumed an “alternating pancake” configuration.^57^ Probably, the increased oil fraction flowing in the microchannel and the higher production of droplets with respect to the previous conditions cause the build-up of pressure in the main channel, which prevents the droplets from flowing in a single row along the center line of the channel, thus forcing them to organize into an alternating arrangement like a sinusoidal shape.

**Figure 3:**
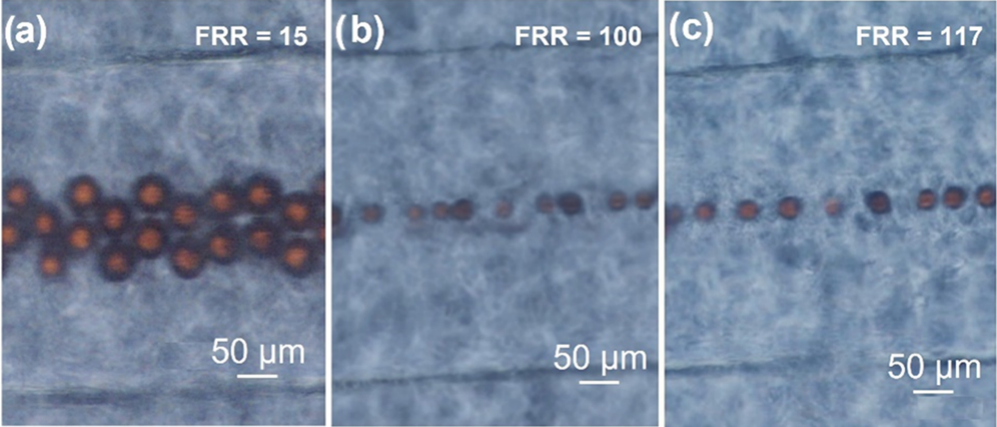
Images showing droplets of (a) SSY 7% wt in water dispersed into the paraffin oil in the presence of Span80 4% wt at FRR = 15, (b) SSY 30% wt in water dispersed into the paraffin oil in the presence of Span80 8% wt at FRR = 100, and (c) SSY 30% wt in water with the addition of Trans-Hyp 26% dispersed into the paraffin oil in the presence of Span80 8% wt at FRR = 117.

Assured that the droplet production of SSY compound is possible, we choose the SSY concentration at 30% wt, which is characterized by a nematic chromonic liquid crystal phase and an increased viscosity. In fact, to observe the stream from breaking, we needed to add to the oily matrix an 8% wt of the surfactant Span80. The increase in surfactant quantity is necessary to maintain the balance between the viscous force and the interfacial tension;^58^ the breaking of the streams was observed with the flow rates of the two fluids set on μL/h for the aqueous phase (SSY) and μL/min for the oily phase (paraffin oil + 8% wt Span80). An FRR in the interval 100–117 was used to produce droplets with diameters ranging between 10 and about 50 μm (Figure S2); no coalescence effects were observed (Figure 3a). The same behavior was observed for the production of SSY microdroplets when the solution was doped with Trans-Hyp at 16% wt and 26% wt (Figure 3b,c). The Span80 concentration was kept constant, indicating that the addition of the chiral dopant does not affect droplet production probably because it does not alter significantly the equilibrium between viscosity forces and interfacial tension.

**Figure 4:**
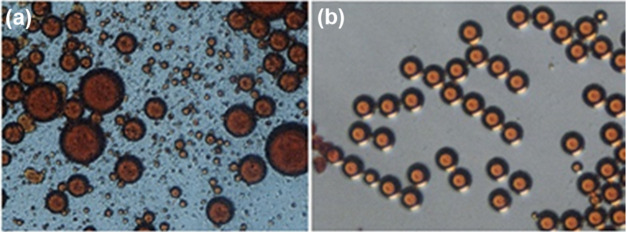
(a) Coalescence observed for microdroplets of pure 7% wt SSY produced using 4% wt Span80 added to the oily matrix and (b) SSY 7% wt in water dispersed into the paraffin oil in the presence of Span80 8% wt.

Possible coalescence effects that could occur with time were also studied. After microfluidic production, both pure and chiral nematic SSY droplets were collected inside glass vials, which were filled with the same percentage of paraffin oil and surfactant used in the FF device. The time evolution of the droplets was investigated by checking the vials after 24 h. Coalescence effects have been observed only for microdroplets of pure 7% wt SSY produced using 4% wt Span80 added to the oily matrix (Figure 4a), while for isotropic, nematic, and chiral nematic droplets produced using 8% wt Span80, no coalescence effects were observed (Figure 4b). Hence, in addition to helping the microdroplet production, the presence of the surfactant also avoids coalescence effects, ensuring the time stability of the emulsions.

### Optical Characterization of the Microdroplets

Similar to thermotropic LCs, the topologies observed in LCLC microdroplets strongly depend on the droplet diameter, anchoring condition at the boundaries, and, for chiral mixtures, the helical pitch.^2,18,59^ At odds with what happens for thermotropic LCs, controlling the director configuration inside the microdroplets of LCLCs is a rather difficult task. However, the vast literature on the geometrical frustration in the thermotropics can be used to interpret the director field configuration inside the LCLC microspheres. In this perspective, we carried out systematic polarized optical microscopy (POM) measurements on the different microdroplets produced by the microfluidic device using paraffin oil that provides planar anchoring conditions. Droplets were imaged out of the device (after collection in a vial) at first without polarizers to correctly estimate their diameter and then with crossed polarizers. The observed textures were identified by rotating the microscope plate and rotating the analyzer.

**Figure 5:**
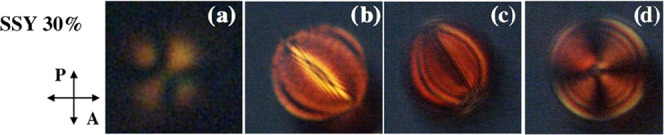
POM images between crossed polarizers of microspheres of pure nematic phase SSY at 30% wt. Droplet diameters are estimated from the bright field image. Specifically, the diameter of the reported microspheres is (a) 10 ± 1 μm; (b) 47.0 ± 1.7 μm; (c) 49.0 ± 1.2 μm; and (d) 52.0 ± 1.4 μm.

Figure 5 shows the size-dependent topologies observed in microdroplets of pure nematic phase SSY. In general, small droplets (10–20 μm) show a bipolar structure (BS) (Figures 5a and S3). This is the typical texture observed in the chromonics microspheres with planar boundary condition^60^ in which the director is parallel to the interface and forms two boojums at the poles. For droplet diameters ranging between 30 and 50 μm (Figure 5b,c), a better-defined bipolar structure was observed. In a few cases, a “concentric drop” texture is observed, which can be related to the top view of a bipolar texture (Figure 5d). In this configuration, the director field is organized in concentric circles with a disclination line that passes through the center of the drop. It is interesting to notice that the surfactant, added to allow the production of stable microfluidic droplets, does not affect the optical properties and textures, thus confirming its purely interfacial role in droplet formation.

**Figure 6:**
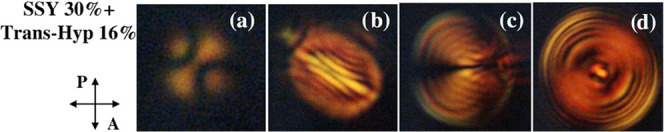
POM images between crossed polarizers of microspheres of SSY at 30% wt + 16% wt of Trans-Hyp. Droplet diameters are estimated from the bright field image. Specifically, the diameter of the reported microspheres is (a) 10 ± 1 μm; (b) 20.0 ± 1.8 μm; (c) 45.0 ± 1.7 μm; and (d) 50.0 ± 1.5 μm.

Figure 6 shows a collection of SSY microspheres doped with Trans-Hyp at 16% wt (Figure S4). Small droplets (10–20 μm diameter) show bipolar configuration, as in the case of the pure nematic phase (Figure 6a,b). The addition of the chiral dopant is reflected in the optical texture observed in the microdroplets. In fact, for a droplet diameter larger than 45 μm, the radial spherical structure (RSS) develops (Figure 6c). In the RSS, the director profile is characterized by a splay-bend distortion inside the droplet that generates a disclination line, similar to the so-called Frank–Pryce structure observed in thermotropic LCs. Increasing the droplet diameter, another topology was observed. It could be related to the diametrical spherical structure (DSS) or the top view of the RSS (Figure 6d). The DSS is the most symmetric structure in cholesteric droplets with planar anchoring. It is characterized by a center ring defect and the director field forms curved cholesteric layers with the layer normal in the radial direction.

**Figure 7:**
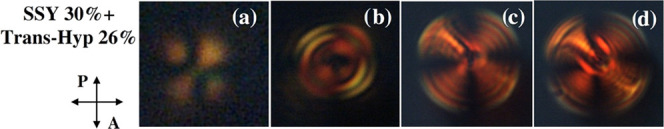
POM images between crossed polarizers of microspheres of SSY at 30% wt + 26% wt Trans-Hyp, collected according to their dimensions. Droplet diameters are estimated from the bright field images. Specifically, the diameter of the reported microspheres here is (a) 10 ± 1 μm; (b) 20 ± 2 μm; (c) 50.0 ± 1.5 μm; and (d) 57.0 ± 1.6 μm.

Figure 7 shows the POM images of SSY microspheres doped with 26% wt of Trans-Hyp at different droplet diameters (Figure S5). As observed for the other doped solutions, small droplets with a diameter of about 10 μm (Figure 7a) show a bipolar configuration, while the most interesting topologies are observed for droplets with increasing diameters. For diameters around 20 μm, the texture starts to become distorted in radial concentric rings (Figure 7b). This effect is more evident in Figure 7c in which a clear Frank–Pryce texture is shown. In Figure 7d, we can distinguish a topology, observed only a few times, that resembles the Lyre structure and is numerically predicted by Seč et al.^61^ in cholesteric thermotropic LC droplets. The Lyre structure, of bipolar type, has only two surface defects positioned diametrically along one of the axes parallel to the observation plane. This is the first experimental observation of this kind of structure in an LCLC droplet probably due to its metastable nature.

In Table 1, we collected schematically the director configuration observed inside the droplets associated with the microdroplet diameter for each LCLC solution studied in this work. Small droplets (diameters 10–20 μm) show a bipolar configuration for both pure and chiral doped solutions. The distortion increases both with the microsphere’s diameter and the concentration of the chiral agent. Even if the material used is a chromonic mesogen, the trend observed for the optical textures is in agreement with the reported data in the literature on the diameter/topology dependence in thermotropic nematic and chiral LCs.

**Table 1:** LCLCs Microdroplet Optical Textures Reported for Each Droplet Dimension and Each Investigated Liquid Crystal Solution^a^

	SSY 30%	SSY 30% + 16% Trans-Hyp	SSY 30% + 26% Trans-Hyp
bipolar structure, BS	10–50 μm	10–20 μm	∼10 μm
RSS		>45 μm	20 μm
DSS		50 μm	50–60 μm
Frank–Pryce			50 μm

a The percentage of SSY and Tran-Hyp indicated in the figure are in weight percentage (%wt).

## Conclusions

The confinement of self-assembling materials, especially chiral liquid crystals, in spherical micro-objects with a controllable size is crucial to obtain reproducible optical patterns that can be used for practical applications.

Here, we report on the microfluidic production of microspheres containing a chromonic liquid crystal, Sunset Yellow. Exploiting and characterizing the interplay of the interfacial phenomena, surface effects, and geometric confinement, we were able to define a protocol to produce controlled size and topology of nematic and chiral SSY microdroplets. Studying the wettability of the channels, we identified a functionalization of the glass device based on silanes to reduce the interactions of the walls with the SSY phase and increase the affinity of the oil phase for the walls, which allowed a correct and stable flow in the flow-focusing device. The microfluidic SSY microdroplets show unique optical topologies, like the ones observed with thermotropic LC microdroplets in planar boundary conditions. This is a relevant achievement considering that the literature presents just a few works on the microfluidic production of nematic SSY chromonic microdroplets and no data are available on chiral SSY microspheres. From a fundamental point of view, this can help to better understand the interactions among the supramolecular structures, studying, for example, the effect of charged guest molecules on the optical patterns.

Doping the chromonic material with chiral amino acids has allowed the study and production of structures and topologies that are typically observed in cholesteric thermotropic LC microdroplets. In chiral SSY microdroplets, a structure never observed before and similar to that of Lyre was observed. This is a first step toward the goal of obtaining chromonic microspheres with homogeneous tunable optical properties, such as reflection selectivity, which could open new perspectives for the use of these biocompatible materials as sensors and optical devices.

## Experimental Section

### Materials and Solution Preparations

Sunset Yellow (SSY, Sigma-Aldrich) was used as received. At room temperature, it is a red powder and shows a nematic phase if dissolved in water above 28% in weight. Chirality is induced doping the SSY with a proper amount of *trans*-4-Hydroxy-l-proline (Trans-Hyp) from Sigma-Aldrich. Paraffin oil and Span80 were purchased from Sigma-Aldrich. A mixture of paraffin oil and Span80 was used as an oil matrix in the microfluidic device. Nonchiral SSY liquid crystal mixtures were prepared using deionized water (18.2 MΩ cm) to make a solution of known concentration and phase (30% wt for SSY). For chiral mixtures, we added 16% wt and 26% wt Trans-Hyp (dissolved in water) to 30% wt SSY.

### Microfluidic Device Fabrication and Experimental Setup

The FF droplet generator was a glass-based device with an overall dimension of about 2.5 × 5 cm^2^. For the fabrication of microchannels, commercially available B-270 glasses, covered with a 450 nm thick chromium layer (Telic), were used as solid substrates. Hydrochloric acid (HCl), ammonium fluoride (NH4F), and hydrofluoric acid (HF) were purchased from Sigma-Aldrich (Taufkirchen, Germany). The resist AZ9260 and the AZ400k developer were purchased from MicroChemicals (Ulm, Germany). The chromium etchant solution was purchased from Sigma-Aldrich. Photomasks were designed using CleWin software and printed by J. D. Photo-tools Ltd. (Oldham, Lancashire, U.K.). Fluoropolymer tubings (Tub FEP Blu 1/32 × 0.09) were purchased from IDEX Health & Science (Germany). The microfluidic network reported in the scheme of Figure 1 was patterned on a B-270 glass substrate via photolithography. After the geometry transfer, the glass substrate was etched with buffered oxide etchant (BOE) solution by using the microwave reactor system (Anton Paar Multiwave 3000, Labservice Analytica s.r.l., Italy) as reported in ref (26) for obtaining a channel depth of 100 μm.

Then, three holes were processed by using a microdriller (MICRO miller MF70, Proxxon, Germany) in order to create two inlet ports and one outlet port. The channel was then thermally bonded to a glass top plate,^62^ and finally, capillary tubes were connected with the inlet and outlet holes. After the fabrication, the internal walls of the microchannels were functionalized with a hydrophobic coating. The microchannels were filled with 1H,1H,2H,2H-perfluorooctyltriethoxysilane at a constant rate of 30 mL/min using a syringe pump (Ugo Basile, Biological Research Apparatus, model KDS270). After complete filling, the silane was incubated for 30 min and then withdrawn at 150 mL/min. As the final step, the microchannel was dried by pumping air. This procedure assures that the channel walls are covered by the silane layer as reported in ref (63).

After functionalization, the device was used to produce the microdroplets by injecting paraffin oil with Span80 (4% wt and 8% wt) from the side channels and the aqueous solution of pure SSY (7% wt and 30% wt) and chiral doped SSY 30% wt (chiral agent at 16% wt and 26% wt) from the central inlet. The flow rates of continuous and dispersed phases were controlled by two independent pumps (model KDS270).

Images and videos were acquired by a NIKON mod. DS-5MC camera with an 8 fps acquisition rate. After flowing through the microfluidic network, droplets were collected and analyzed on a cleaned glass slab by an optical microscope (Nikon Eclipse Ti) equipped with polarizers. The sizes of the droplet and image analysis were performed through Nikon Eclipse software within the device (Figures 2 and 3) and through ImageJ software for characterizations out of the device (Figures 5–7).

### Surface Functionalization and Contact Angle Measurements

**Figure 8:**
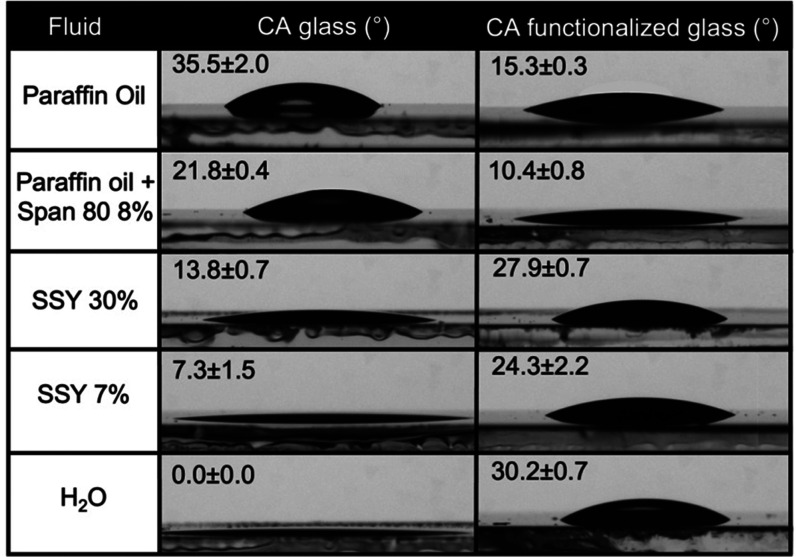
Contact angles of the different fluids on glass and functionalized glass.

To assess the efficacy of the functionalization with the silane in increasing the affinity of the oil phase for the device walls and decreasing the affinity of the water phases, as well as to understand the role of surfactant Span80 in the interactions between the oil phase and the functionalized walls, some contact angle measurements have been performed. For this purpose, B-270 glass substrates were treated with the BOE solution and thermal process, by following the same procedure used for the microfluidic device fabrication. To coat the glass slice with a silane layer, a drop of 1H,1H,2H,2H-perfluorooctyltriethoxysilane was deposited on the substrates, incubated for 30 min, and finally dried with nitrogen.^64^ After preparation, contact angle (CA) measurements were acquired using the sessile drop method with the CAM 200 instrument (KSV Instruments Ltd., Finland). Several drops of paraffin, paraffin with Span80 (8% wt), and SSY in water at 7% wt and 30% wt (isotropic and nematic phase) were deposited onto different areas of bare and functionalized glass substrates (Figure 8). The respective averages of the CA values are reported in Table 2. The errors are calculated as standard deviations from the average value.

**Table 2:** Contact Angle (CA) Measurements of the Fluids Used for the Microfluidic Droplet Generation on Bare and Functionalized Glass Substrates^a^

fluid	CA on glass (deg)	CA on functionalized glass (deg)
paraffin oil	35.46 ± 2.04	15.31 ± 0.30
paraffin oil + Span80 8%	21.84 ± 0.35	10.40 ± 0.81
SSY 30%	13.79 ± 0.68	27.85 ± 0.66
SSY 7%	7.33 ± 01.54	24.26 ± 2.16
H_2_O	0	30.21 ± 0.65

a The functionalization of the glass results in a higher CA for paraffin oil and paraffin oil + Span80 8% wt, and a lower CA for SSY (30% wt and 7% wt) and water. The percentage of Span80 and SSY in the table are in weight.

The CA values on the clean and functionalized glass substrates demonstrated that the glass walls of the device are covered by a silane layer that enhances the surface hydrophobicity as shown by the water contact angle measurements of the clean and functionalized glass substrate, reported in Table 2. This fact increases the affinity of the oil phase for the walls and decreases the affinity of both water-based nematic (SSY 30% wt) and isotropic (SSY 7% wt) solutions. The affinity of the paraffin oil for the functionalized walls is stronger, implying that, when the Span80 surfactant is added, part of it is involved in the walls/oil interaction and it is not available at the oil/water interface of the droplets. Thus, a large amount of surfactant must be added to compensate for the fraction subtracted by the wall/oil interface; this can explain why the amount of surfactant we use is double and quadruple, respectively, with respect to the typical concentrations with a similar setup.^45^

## Supporting Information Available

Droplets diameter as a function of the FFR. Topologies observed in nematic and chiral chromonics (PDF)
