# Supplementary material for: Sunset Yellow Confined in Curved Geometry: A Microfluidic Approach
Source: Langmuir. 2023 Apr 19;39(17):6134–41. doi: 10.1021/acs.langmuir.3c00275 (PMC10157883; doi:10.1021/acs.langmuir.3c00275)
Supplement: Supplementary file 1 — la3c00275_si_001.pdf [file la3c00275_si_001.pdf]

Supporting Information

Sunset Yellow confined in curved geometry: a microfluidic approach

Caterina Maria Tone[1,2]*, Alessandra Zizzari[3]*, Lorenza Spina[1,2], Monica Bianco[3], Maria Penelope De Santo[1,2]*, Valentina Arima[3]*, Riccardo Cristoforo Barberi[1,2] and Federica Ciuchi[2]

[1]*Physics Department, University of Calabria, Ponte P.Bucci, cubo 31C, Arcavacata di Rende, 87036 (CS), Italy*
[2] *CNR-Nanotec c/o Physics Department, University of Calabria, Ponte Bucci, cubo 31C, Arcavacata di Rende, 87036 (CS), Italy*
[3] *CNR NANOTEC-Institute of Nanotechnology, c/o Campus Ecotekne, University of Salento, via Monteroni, 73100 Lecce, Italy*

Corresponding authors

caterina.tone@fis.unical.it; maria.desanto@fis.unical.it valentina.arima@nanotect.cnr.it; alessandra.zizzari@nanotec.cnr.it

Table of contents

Figure SI1 Histogram of droplets diameter distribution Pag S2

Figure SI2 Droplets diameter as a function of the FFR Pag S2

Figure SI3 POM images of pure SSY30%wt Pag S3

Figure SI4 POM images of SSY30%wt+Trans-Hyp 16%wt Pag S3

Figure SI5 POM images of SSY30%wt+Trans-Hyp 26%wt Pag S3

Droplets diameter as a function of the FFR.

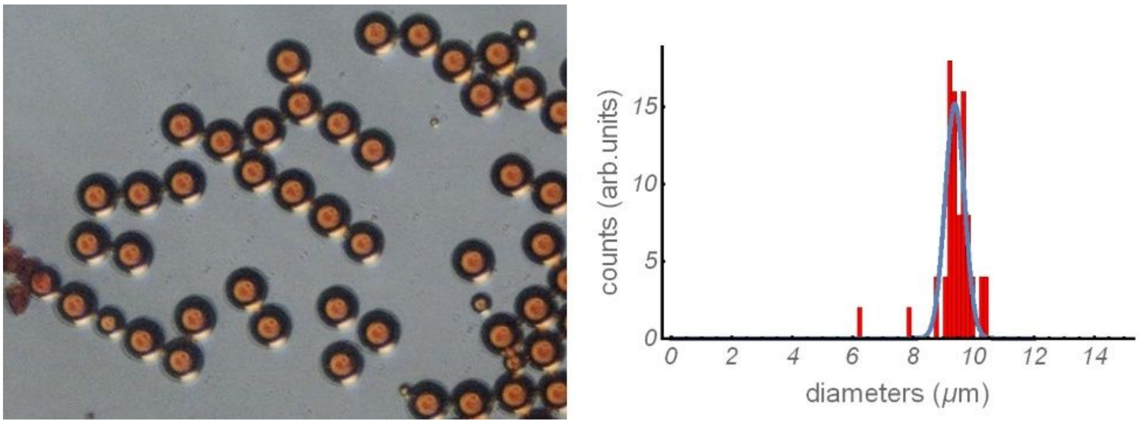

Figure SI1. SSY7% microspheres produced using Paraffin oil +8%wt. Span80 (left). Histogram of the measured diameters fitted with a Gaussian curve (right).

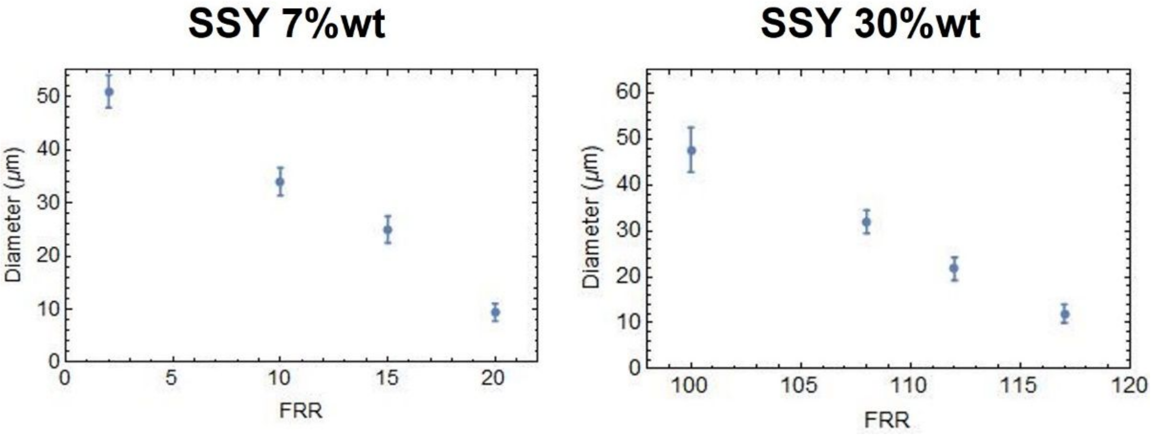

Figure SI2. For both SSY concentration in paraffin oil + 8%wt. Span80 the diameter as a function of FRR was measured.

Topologies observed in nematic and chiral chromonics

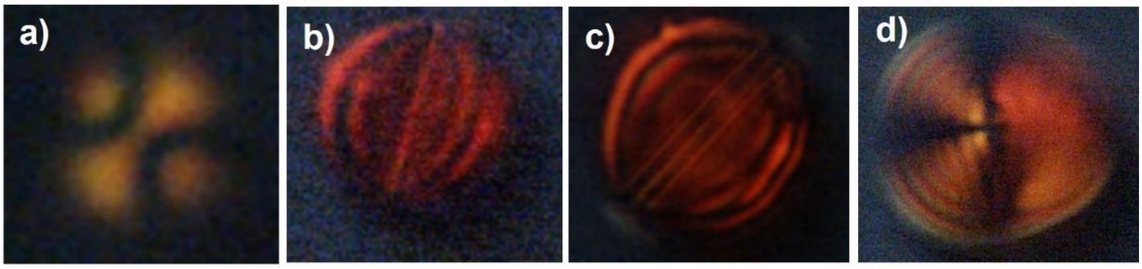

Figure SI3. POM images between crossed polarizers of microspheres of pure nematic phase SSY at 30%wt. The diameters of the reported microspheres are: a) (10.0±0.8) µm; b) (34.0±1.8) µm; c) (40.0±1.4) µm; d) (49.0±1.0) µm.

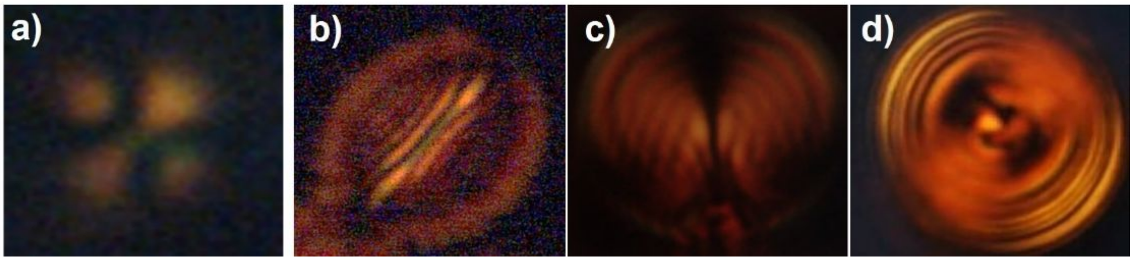

Figure SI4. POM images between crossed polarizers of microspheres of SSY at 30%wt. + 16%wt. of Trans-Hyp. Specifically, the diameters of the reported microspheres are: a) (10.0 ± 1.0) µm; b) (32.1 ± 1.8) µm; c) (44.3 ± 1.9) µm and d) (49.0 ± 2.0) µm.

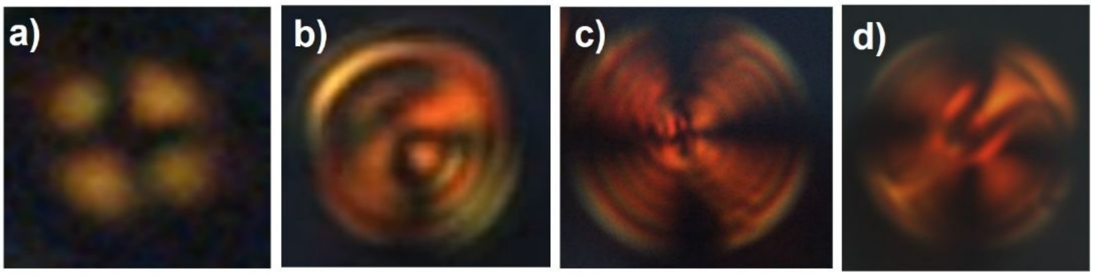

Figure SI5. POM images between crossed polarizers of microspheres of SSY at 30%wt. + 26%wt. of Trans-Hyp. Specifically, the diameters of the reported microspheres are: a) (10.2 ± 1 .0µm); b) (21.0 ± 1.3 µm); c) (54.0 ± 1.0 µm) and d) (56.2 ± 2.0) µm.
